# Factors affecting recruitment into depression trials: systematic review and meta-synthesis of qualitative evidence

**DOI:** 10.1186/1745-6215-14-S1-P82

**Published:** 2013-11-29

**Authors:** Adwoa Hughes-Morley, Bridget Young, Peter Bower

**Affiliations:** 1 MRC North West Hub for Trials Methodology Research, Centre for Primary Care, Institute of Population Health, The University of Manchester, Oxford Road, Manchester, UK; 2 MRC North West Hub for Trials Methodology Research, Institute of Psychology, Health and Society, University of Liverpool, Liverpool, UK

## Background

Depression is common, and clinical trials are crucial for evaluating treatments. Difficulties in recruiting participants into depression trials are well-documented, yet no study has comprehensively examined the factors affecting recruitment. The objective of this review is to identify the factors affecting recruitment into depression trials through systematic assessment of published qualitative research.

## Methods

Systematic review and meta-synthesis of published qualitative studies. Meta-synthesis reconceptualises themes across a number of qualitative studies to combine phenomena into a transformed whole. Medline, Embase, PsychInfo and CINAHL were searched up to April 2013. Reference lists of included studies, key publications and relevant reviews were also searched. Quality appraisal adopts the minimally prescriptive “Prompts for appraising Qualitative Research”, combined with appraisal of contribution and credibility during the synthesis process (Dixon-Woods, 2006).

## Results

7977 citations were identified, and 19 studies are included. Preliminary themes in the identified studies include both barriers and facilitators. Barriers include endorsement of depression symptoms; stigma; the difficulty of introducing the idea of research in a consultation involving depression; protecting the vulnerable patient; and trial related issues (trial burden, treatment preferences, and randomisation). Facilitators include altruism; practical support for participation; access to services to meet mental health needs; clear processes, and; regular feedback to recruiting clinicians. The meta-synthesis (in progress) will seek to develop a broader explanatory framework to better understand barriers to participation.

## Conclusions

It is hoped that the explanatory framework will enable the development and evaluation of recruitment strategies that address these factors to improve recruitment into depression trials.

